# Kidney Disease in the Insane: A Study of Six Hundred Urinalyses and Seventy Autopsies

**Published:** 1902-06

**Authors:** M. L. Perry

**Affiliations:** Pathologist to the Georgia State Sanitarium, Milledgeville, Ga.


### KIDNEY DISEASE IN THE INSANE: A STUDY OF SIX HUNDRED URINALYSES AND SEVENTY AUTOPSIES.*

By M. L. PERRY, M.D.,

Pathologist to the Georgia State Sanitarium, Milledgeville, Ga.

Within the past few years a great deal has been written upon the subject of diseases of the kidneys in the insane. By far the greater

*Read before the Georgia Medical Association, Savannah, April, 1902.

part of this literature, however, has been devoted to the discussion of a single disease of these organs, viz.: nephritis. Most of the articles treating of this subject have been purely clinical, being based upon a study of the urine, or have dealt entirely with conditions found *post mortem.* Now there can be no doubt but that looking at disease from one side only, be it as a clinician or as a pathologist, is very prone to warp the judgment and lead to error. So, believing a combination of the two methods to be a more satisfactory and reliable way to study the question, I present below a synopsis of six hundred urinalyses made in the laboratory of the State Sanitarium during the past two years, together with notes on the kidneys of cases coming to autopsy during the same period. It is our routine practice to examine the urine of each patient entering the institution as soon as possible after admission, and it is from the records of these examinations that most of my data has been taken. The urinalyses, therefore, refer entirely to new patients. The urine is examined both chemically and microscopically. It is tested chemically for albumin, indican and acetone, and the microscopical examination is made of the sediment thrown down with the centrifuge.

I have not been able to compute the total urinary solids eliminated by each of these six hundred patients. Such information would certainly be of the greatest interest and importance if it could be obtained, but a moment's thought will show that it is quite impossible to get it. To reckon the total solids one must have the entire urinary secretions for twenty-four hours. This is easy enough in general practice where one has the co-operation of the patient, but in the insane it is a very different matter. It is often difficult, owing to the maniacal state of a patient or to the development of delusions upon the subject, to get even a few ounces of urine for examination, and under these circumstances the total amount is out of the question. I have, however, recently selected fifty cases whom I could control, and have in these calculated the total solids excreted by the kidneys for two successive days, taking the mean as the amount for twenty-four hours. Of these cases some were acute, but the greater part were of a chronic type. They were selected so that a number of different forms of insanity would be represented in the group. The result of this investigation was indeed surprising. In 90 per cent. of the acute cases studied the total quantity of urinary solids excreted was diminished. In some instances it was less than one-third the normal amount. In the chronic cases exactly 50 per cent. showed a diminution of the total solids. An examination of the urine failed to reveal any evidence of nephritis in many of the cases where the solids were diminished. While the number of cases under observation was comparatively small, the results were sufficiently positive to enable us to say that functional derangement of the kidneys is undoubtedly present in a large per cent. of the insane.

The following is a synopsis of urinalysis: As to the total amount of urine passed in twenty-four hours, I can give no definite figures, but from close observation can say positively that in the majority of patients the urine is diminished in quantity. This is especially true of acute cases, in whom it is oftentimes reduced to one-fourth the normal.

The specific gravity as might be expected, since the urinary amount was diminished, was found to be above the normal in 60 per cent. of all cases.

CHEMICAL ANALYSIS.

| | | | | | |
|---|---|---|---|---|---|
| Indican | present | in | 55.1 % | of | cases. |
| Acetone | " | " | 4.6 % | " | " |
| Serum albumin | " | " | 28.1 % | " | " |
| Serum albumin with no casts in sediment | " | " | 7.8 % | " | " |

MICROSCOPICAL EXAMINATION.

| | | | | | |
|---|---|---|---|---|---|
| Amorphous and crystalline urates | present | in | 14.0 % | of | cases. |
| Uric acid crystals | " | " | 2.3 % | " | " |
| Calcium oxalate crystals | " | " | 7.0 % | " | " |
| Casts | " | " | 40.0 % | " | " |
| Casts without albuminuria | " | " | 19.1 % | " | " |
| Casts in connection with kidney epithelium or albuminuria | " | " | 33.7 % | " | " |

From the foregoing statement it will be seen that the most frequently found abnormal substance in the urine of these patients was indican. I have been careful not to include in these statistics cases which yielded only a trace of indican, since that could hardly be considered pathological. In most cases the indican was pronounced. After a careful study of the matter I have found it impossible to associate this condition of indicanuria with any particular form of mental disease or definite mental state. There can be no doubt, however, of its intimate association with insanity in general. It can be taken as an index of the amount of intestinal fermentation and putrefaction present, and would thus indicate that these morbid states exist in a large per cent. of the insane.

The following table gives the form of mental disease of those patients having indicanuria at the time of admission:

| | |
|---|---|
| Mania, acute | 93 |
| Mania, chronic and recurrent | 72 |
| Melancholia, acute | 48 |
| Melancholia, chronic | 25 |
| Dementia, terminal | 7 |
| Dementia, primary | 2 |
| Paresis | 5 |
| Paranoia | 8 |
| Senile insanities | 14 |
| Pubescent insanity | 5 |
| Toxic insanity | 7 |
| Epilepsy | 31 |
| Imbecility | 14 |
| Total | 331 |

In considering this, and also the similar table relating to Bright's disease, two facts should be borne in mind. First, that I have necessarily had to follow the old asylum practice of differentiating the acute from the chronic cases by a time limit; all of less than twelve months' duration being termed acute. Secondly, that we receive a relatively larger number of acute cases than do most asylums. This is due to the fact that since the institution has been declared full and patients are received only as vacancies occur, admissions have been, as nearly as possible, restricted to the acute and curable class.

I was led to study the subject of acetonuria by a report of some work along this line by Von Wagner, of Vienna. He claimed that the urine of patients suffering with certain acute psychoses, especially acute delirium, was loaded with acetone, and that it might be taken as an evidence of intoxication. I have not been able to get any such result, and I do not consider that acetonuria has any special relation with mental disease. I have found it in only a small number of cases, and some of my patients in whom it was marked have been of the milder forms of insanity.

The synopsis given above shows some interesting facts in regard to nephritis. I have considered as cases of Bright's disease those cases only which have shown casts in the urine in connection with kidney epithelium or albuminuria. Thirty-three and seven-tenths per cent. of all the specimens examined have come under this class. Here, as with indicanuria, I have not been able to associate it with any particular form of psychosis. The following table gives the form of mental disease of those patients having Bright's disease at the time of admission:

| | |
|---|---|
| Mania, acute | 59 |
| Mania, chronic and recurrent | 52 |
| Melancholia, acute | 25 |
| Melancholia, chronic | 15 |
| Dementia, terminal | 4 |
| Dementia, primary | 1 |
| Paresis | 7 |
| Paranoia | 5 |
| Senile insanities | 15 |
| Pubescent insanity | 2 |
| Toxic insanity | 2 |
| Epilepsy | 12 |
| Imbecility | 3 |
| Total | 202 |

The relation existing between casts and albuminuria deserves, I think, some special notice. It will be seen above that 7.8 per cent. of the specimens examined contained serum albumin without casts, and 19.1 per cent. showed the presence of casts without albumin. The first of these conditions is nothing very unusual, as transitory or physiological albuminuria is often met with. But that so high a per cent. of cases should show casts in urine entirely free from albumin is quite extraordinary. Considerable difference of opinion still exists among authors as to the nature and origin of casts. Purdy gives as the three chief views on the subject, first, that they are the result of disintegration of the epithelium of the renal tubules; second, that they consist of a secretion of the morbidly irritated epithelium lining the renal tubules, and third, that they consist of the coagulable elements of the blood which gains access to the renal tubules through pathological lesions of the latter. He states further that the last view is the one most generally accepted, at least so far as the nature and origin of most casts are concerned. The supporters of this last view, while admitting that casts are sometimes found without albumin, claim, notably Purdy and Bartels, never to have met with them save in albuminous urine or urine that has recently been albuminous. The fact that so large a per cent. of my cases showed casts without the presence of albumin can hardly be reconciled with this last theory. As further evidence that casts cannot always be accounted for in this way, I will state that I have in several cases saved the entire urinary secretion and examined it every day for a week at a time, finding casts at each examination and albumin at no time. In these experiments I have resorted to several tests in addition to the ferrocyanic test, which is the one usually relied upon. From these facts I am more inclined to look upon casts in the urine as more probably a secretion of the morbidly irritated renal epithelium. In substantiation of this it may be mentioned that cylindroids, which are generally accepted to be the result of irritation, were found in a large number of my cases. Taking this view as the correct one, we would here have evidence of the presence in the circulation of a decided irritant to the renal epithelium. But whichever theory we accept, the result of these urinalyses must necessarily be taken as further substantiation of the statements of other writers upon the subject as to the prevalence of Bright's disease in patients suffering with mental disease.

The autopsies comprised in this study are the first seventy cases on my autopsy records, and have been taken in regular order, no selection of special cases having been made. They are, with a few exceptions, negroes. Their ages range from fifteen to seventy-five years; about one-half being under thirty-five. An analysis of the mental condition of these cases will show that most of the common forms of insanity are represented. As regards the duration of their mental disease, some were acute, but the larger number were of a chronic type. They died from various diseases, but in only a few instances was any disease of the kidney given as a cause of death. They may, I think, be taken as fair representatives of asylum patients in general.

A study of the kidneys of these cases showed that there were two pathological conditions which were present in a large per cent. of the total number. These two diseases are, in the order of their frequency, arteriosclerosis and chronic nephritis. The former, arteriosclerosis, was present in 88.5 per cent. of all cases. The extent to which the vessels were affected varied, of course, in different cases. In some the change was limited almost entirely to the adventitia, and the caliber of the vessel was not seriously constricted. In others, however, and this was true of the most of them, all the coats were involved and the blood supply was necessarily much diminished. In fact, in 71 per cent. of the total number this condition had progressed to such an extent that sections made from the kidney showed malpighian bodies in which the tufts of capillaries had been completely destroyed and replaced by balls of functionless and useless fibrous tissue. This lesion was in nowise limited to patients well, or even moderately, advanced in years, for it was found in a considerable number under thirty years of age; and in two cases of twenty-one years and one of fifteen years there was pronounced arteriosclerosis with degeneration of capillary tufts. I know of no statistics dealing with the prevalence of arteriosclerosis in general, but there can be no doubt of the fact that the disease exists to a much greater extent in the insane than in the sane. I believe it is also true that in no other class of patients is it so often found in the early years of life.

Turning from disease of the vessels to the other important lesion found, viz.: nephritis, we meet an array of facts quite as extraordinary and of equal clinical importance. Of the whole number of my cases, 67 per cent. showed nephritis sufficiently advanced to be determined by the gross appearance of the kidney, and upon microscopical examination of those kidneys which did not present gross lesions the morbid process, in a less advanced state, was found in enough more to bring the total number of cases of Bright's disease up to 85.5 per cent. The gross appearance of those kidneys which were the seat of nephritis showed considerable variation in size, shape and color. The lowest weight of the two kidneys in any case was 132 gms., and the highest was 570 gms. The majority, however, were under the normal in size and weight. Taking 297 gms. as the normal weight of the two organs in the male, and 269 gms. as the normal weight in the female, and these are figures given in standard works, 70 per cent. of the diseased kidneys fell below the normal.

On section the most frequent macroscopical changes observed were more or less obliteration of the cortical striations and fatty degeneration of the cortex. These conditions were present in a marked degree in most of the specimens.

On microscopical examination there was found in every case a considerable involvement of each of the three tissues entering into the make-up of the kidney, viz., the vessels, tubules and fibrous connective tissue. The pathological changes were distributed with more or less severity throughout the organ, but it is of special interest to note that the parts most seriously affected were almost invariably in the vicinity of the vessels. A general involvement of the structural tissues is usually found in nephritis, and authors of text-books on pathology classify the different types of the disease according as one or the other of these tissues are most extensively affected. Thus, we have the parenchymatous form, where the epithelium has suffered most; the interstitial, in which the connective tissue change is most pronounced; and the glomerular type, if the capillary tufts are chiefly involved. Fortunately for the practical worker there is given still another class, the diffuse, which includes all cases that do not fit elsewhere. That the clear-cut and distinct classification of Bright's disease into the various types as given by some writers is better adapted to the text-book than to the laboratory, must be apparent to all who have done much laboratory work. These facts regarding classification are mentioned to explain why it has been impossible for me to give any statistics on the various forms of nephritis found. In my cases there was every gradation from the type in which the parenchyma certainly presented the chief involvement, to those which might well be described as interstitial nephritis, while in almost all there was more or less destruction of glomeruli. Ziegler, in discussing the two extreme types, says: "In some instances interstitial nephritis differs from the chronic parenchymatous variety rather in degree than in kind." I am inclined to look upon these different pathological states found in my own cases as representing different stages of the same disease, rather than various types. The pathological process may be described as a primary, chronic, progressive, degeneration of the parenchyma, accompanied by a very low form of inflamma-tion of the other structures of the kidney. That the disease is primary and not secondary to acute nephritis may be inferred from the fact that acute nephritis is not of frequent occurrence in asylum practice, and the figures above show that a large number of patients contract chronic disease of the kidney after coming to the institution.

A comparison of the relative number of cases showing nephritis at the time of admission, 33.7 per cent., with that found at autopsy, 85.5 per cent., would further indicate that it is not so important a factor in the etiology of insanity as has been supposed, but rather that it develops along with the latter disease.

The more salient facts brought out by the above study may be summarized as follows:

1. That diseases of the kidney are much more frequent in the insane than in the sane.

2. That the morbid states of the kidney associated with insanity include both functional and organic diseases.

3. That the functional derangements are more pronounced in the acute psychoses, and the organic lesions are more prevalent in cases of long standing.

4. That in both acute and chronic forms of insanity there is marked evidence of irritation of the renal epithelium.

5. That nephritis is not so important a factor in the etiology of insanity as has been supposed.

6. That a study of the kidney and the urine in the insane gives positive evidence of intoxication in a large per cent. of cases.

